# Lightweight PVIDNet: A Priority Vehicles Detection Network Model Based on Deep Learning for Intelligent Traffic Lights

**DOI:** 10.3390/s20216218

**Published:** 2020-10-31

**Authors:** Rodrigo Carvalho Barbosa, Muhammad Shoaib Ayub, Renata Lopes Rosa, Demóstenes Zegarra Rodríguez, Lunchakorn Wuttisittikulkij

**Affiliations:** 1 Department of Computer Science, Federal University of Lavras, Minas Gerais 37200-000, Brazil; rodrigo@estudante.ufla.br (R.C.B.); renata.rosa@ufla.br (R.L.R.); demostenes.zegarra@ufla.br (D.Z.R.); 2 Department of Electrical Engineering, Chulalongkorn University, Bangkok 10330, Thailand; 6271015521@student.chula.ac.th

**Keywords:** intelligent traffic light, deep learning, image detection, vehicle classification

## Abstract

Minimizing human intervention in engines, such as traffic lights, through automatic applications and sensors has been the focus of many studies. Thus, Deep Learning (DL) algorithms have been studied for traffic signs and vehicle identification in an urban traffic context. However, there is a lack of priority vehicle classification algorithms with high accuracy, fast processing, and a lightweight solution. For filling those gaps, a vehicle detection system is proposed, which is integrated with an intelligent traffic light. Thus, this work proposes (1) a novel vehicle detection model named Priority Vehicle Image Detection Network (PVIDNet), based on $YOLO_{V3}$, (2) a lightweight design strategy for the PVIDNet model using an activation function to decrease the execution time of the proposed model, (3) a traffic control algorithm based on the Brazilian Traffic Code, and (4) a database containing Brazilian vehicle images. The effectiveness of the proposed solutions were evaluated using the Simulation of Urban MObility (SUMO) tool. Results show that PVIDNet reached an accuracy higher than 0.95, and the waiting time of priority vehicles was reduced by up to 50%, demonstrating the effectiveness of the proposed solution.

## 1. Introduction

Traffic congestion is a worldwide problem because it affects not only a large part of the population, but also the economy, through delays in the delivery of goods and fuel consumption, causing an inability to estimate travel time [1]. Additionally, the traffic congestion can generate health problems due to the pollution of the gases emitted by cars as well as physical problems of conductors due to the amount of hours spent in the same position inside a car. Thus, maintaining a good vehicular traffic flow seriously impacts people’s quality of life and even safety [2]. For these reasons, studies [3,4] have proposed solutions to reduce congestion in large urban centers, and these solutions propose improvements in urban infrastructure, installing different traffic signs where previously they did not exist.

With the aim to reduce congestion in large cities, relevant research and the emergence of new technologies, such as the evolution of vehicles [5], has occurred in the last several decades. It is important to note that not only the vehicles but also the cities are changing following this evolution. The concept of smart cities is emerging; the infrastructure of cities is becoming smarter and interconnected [6]. These cities use various mechanisms of intelligent infrastructure aimed at the well-being of the population.

Nowadays, autonomous vehicles have been developed [7,8] by some companies. In the same way, computer vision solutions using Deep Learning (DL) algorithms for detection and tracking traffic lights have been proposed [9]. Many autonomous vehicles use Artificial Intelligence (AI) algorithms for detecting objects. Algorithms used on these vehicles include Convolutional Neural Networks (R-CNN) [10], the Faster Region-based Convolutional Network method (Faster R-CNN) [11], You Only Look Once (YOLO) [12], and the Single Shot Multibox Detector (SSD) [13]. They are used for the detection of traffic signs, pedestrians, vehicles, and other objects on the road.

In [14], the authors proposed a solution to classify pedestrians, bicycles, motorcycles, and vehicles, and several tests were carried out to train an DL algorithm, reaching an accuracy of 89.53%. In [15], a system was proposed for classifying cars, pedestrians, drivers, and cyclists, achieving a 90% accuracy rate. In both works, DL algorithms for image detection were used. However, the cited works obtained values of accuracy equal to or lower than 90%.

With the advances on autonomous vehicles using these algorithms, the traffic infrastructure in large urban centers has also been modified. Approaches with the use of AI have been used to improve urban traffic; for example, investments on intelligent traffic lights have been made to reduce traffic congestion and traffic accidents [16]. Some of these traffic lights are considered intelligent because of the use of AI algorithms, the capture of images, or the inclusion of sensors. It is important to highlight that solutions based on sensors have additional costs to be implemented.

For capturing and analyze traffic images, it is necessary to work with almost real-time processing. Studies about intelligent traffic light commonly use, in addition to sensors, images to detect different types of vehicles, such as emergency vehicles [17,18]. Due to the relevance of waiting time in traffic to emergency vehicles, commonly, studies propose a traffic light that gives priority to these vehicles through both audible sensors and images. This system, described in [17], captures images of traffic, identifies a vehicle, and estimates its speed and the distance until it arrives at a traffic light. However, the communications through sensors can fail or can generate false data due to potential problems in the equipment and network. Thus, it is important to have a mechanism independent of a communication network between cars and traffic lights. As previously stated, the current solutions based only on image classification do not achieve reliable accuracy.

Many solutions for real-time image detection explored different DL algorithms, and the solution based on the SSD [13] and YOLO [12] architecture models has obtained the best performance results in the recent literature. A detection system of traffic lights was proposed in [19,20], using SSD, and the response time obtained satisfactory results with a high accuracy. Similarly, the use of $YOLO_{V3}$ [21] had a faster processing speed, detecting objects with a high accuracy, and this has been consolidated in the literature. However, in a traffic lights context, where there are many images that need to be processed in real time, there is a necessity to improve the existing current models, reducing further the processing speed without a negative impact on accuracy.

In this context, an improved version of $YOLO_{V3}$ called the Priority Vehicle Image Detection Network (PVIDNet) is proposed in the present research. To this end, a lightweight design strategy for the PVIDNet model is implemented through an improved Dense Connection model, based on [22], using feature concatenation to obtain a high accuracy and using the Soft-Root-Sign (SRS) [23] activation function for reducing the detection processing speed. In addition, a control algorithm for an intelligent traffic light is proposed. The main goal of this control algorithm is to give priority to emergency vehicles in road intersections controlled by traffic lights. In this work, only ambulances, fire trucks, and police cars are considered as emergency vehicles. Hence, the waiting time of these types of vehicles at traffic lights can be reduced, which is relevant in emergency events.

The proposed solution, composed of PVIDNet and the traffic control algorithm, was evaluated using a simulation tool often cited in related works about urban traffic [24,25,26,27,28], the Simulator of Urban MObility (SUMO), in which the vehicular traffic follows the so-called First-in-First-Out (FIFO) principle. The simulation results show that the proposed solution improves traffic control performance, decreasing the waiting time and the total travelling time, especially in emergency vehicles.

The main contributions presented in this paper are summarized as follows:

- A priority vehicle image detection network (PVIDNet) is proposed based on an improved YOLOv3 model using feature concatenation, and it presents a better detection accuracy than the original YOLOv3 and other image processing-based methods.
- A lightweight model is presented, and the SRS activation function reduces the processing time spent detecting vehicles, maintaining a desirable detection accuracy.
- An improved control algorithm for an intelligent traffic light is introduced based on the Brazilian Traffic Code (BTC), and a new proposal regarding the priority of emergency vehicles is considered.
- A new Database (DB) that considers five types of vehicles—ambulances, police cars, fire trucks, buses, and regular cars—was created. Each image has three different angles (right, left, and front), and each one has the same image resolution characteristics. To the best of our knowledge, there is no available DB in the current literature that considers all these characteristics. Note that each country has different models of emergency vehicles.

The basis of PVIDNet optimisation is the use of dense blocks, improved transition blocks, and the SRS activation function. Such characteristics of PVIDNet optimize the backbone network, enhancing the feature propagation and improving the network performance. The accuracy of the machine learning algorithms to classify the Fire Truck, Bus, Ambulance, Police, and regular Car images in the testing phase, considering together the right, left, and front images, reached values higher than 0.95.

Additionally, the proposed solution reduces up to 50% of the waiting time for priority vehicles, compared to the FIFO strategy, which is still used in many related works [29]. This waiting time reduction is very important for emergency events.

The paper is organized as follows: In Section 2, related works regarding the algorithms used for object detection are presented. Section 3 describes the methodology used for obtaining the proposed solution, the proposed model, PVIDNet, and the algorithm for an intelligent traffic light. The results achieved and discussions about the proposed model are presented in Section 4. Section 5 concludes the paper.

## 2. Related Works

In this section, some works related to DL algorithms applied to object detection and urban traffic solutions using different versions of the YOLO algorithm are presented. In addition, some characteristics of the Brazilian traffic code are treated.

### 2.1. Deep Learning Algorithms for Object Detection

In recent years, several architectural models for DL algorithms have been presented for many applications, each one with its particularities and means of use [30,31,32,33,34].

A DL algorithm used in object detection is the Convolutional Neural Networks (CNN) [35], which is used as the base of many models. R-CNN [10] is an algorithm used for object detection using regions of interest. However, the disadvantage of these algorithms is that they need many sections per image, utilizing processing resources [36] for the object detection [37]. The Fast Region-based Convolutional Network method (Fast R-CNN) [38] is another algorithm very similar to R-CNN. However, to perform the object detection task, this model almost needs a low processing time, greatly improving the speed compared to R-CNN [37]. The Faster R-CNN [11] model is another model based on CNN, composed of two shared modules. The first is a region proposal network (RPN) and the second uses the Fast R-CNN [38] detector. Using neural networks with attention mechanisms, the first module indicates to the Fast R-CNN module where to search for regions in the image. The Faster R-CNN is commonly faster than its predecessors. In addition, studies tested the tuning of parameters to improve the performance of Faster R-CNN on vehicle detection [39].

Some models used for vehicle detection are classified as two-stage algorithms. These algorithms provide better accuracy, but the time for the calculation is quite high. Therefore, in the literature aiming at the object detection processing time, some models of one-stage DL algorithms are proposed, such as SSD [13] and YOLO [12]. However, these algorithms do not achieve sufficient accuracy. Thus, there have been some proposals to improve these algorithms [40].

The SSD algorithm model [13,41] has been widely used for object detection. In [19], a solution was proposed to identify and verify the state of traffic lights. This proposal obtained an accuracy lower than 95%, even with small objects. However, some researchers have modified the SSD algorithms for obtaining an improvement in the precision rate of the standard SSD [42], dividing the weights in the classification networks and consequently decreasing the time training of the network [43].

YOLO [12] uses a unified and fast object detection approach. Its processing is not complex, using images in real time at 45 Frames Per Second (FPS) and a mean Average Precision (mAP) of 63.4%. This model learns the general representations of objects in a short period of time, surpassing other detection methods such as R-CNN and Faster R-CNN [11]. YOLO uses the Darknet framework and the ImageNet-1000 data set to train the model. However, YOLO has limitations related to the proximity of the objects in the image [40]. Another limitation is about the proportions of the object; in case they are different in the images used in the training phase, the model finds some position errors, disturbing the object detection [37,44].

The improved model, $YOLO_{V2}$ [45], is an algorithmic model that can identify and classify in real time about 9000 object categories. This model of algorithm can be executed with varying sizes in the input images, thus allowing for an easy exchange of speed and precision. At 67 FPS, $YOLO_{V2}$ obtains mAP results of 76.8% [45]. At 40 FPS, the mAP can reach 78.6%, surpassing the more advanced methods such as Faster RCNN [11] with ResNet and SSD. In the ImageNet validation set, it obtains 19.7% of mAP, whereas, with the Common Objects in Context (COCO) database, it has a mAP of 16.0%, in all validation sets [45]. In general, $YOLO_{V2}$ adopts a set of changes to improve the speed and accuracy when compared to YOLO, and some of these changes are batch normalization, a high resolution classifier, the use of anchors, and training at various scales [45].

$YOLO_{V3}$ [46] has undergone several small changes in its configuration, improving the mAP when compared to other algorithm models, such as SSD or $YOLO_{V2}$. According to [47], $YOLO_{V3}$ is an improved version of $YOLO_{V2}$ with the aim to obtain a higher accuracy through the use of scales forecasts, multi-label classification prediction, and a resource extractor characteristic.

### 2.2. Urban Traffic Solutions Using Different Versions of the YOLO Algorithm

Aiming at public safety, in [48], the authors proposed the detection of pedestrians using YOLO based on the Gaussian Mixture Model (GMM) or the Gaussian mix model. The GMM model is used to subtract the background from the images, modeling the value of each single pixel. In [49], the YOLO-PC model was proposed, and this model consists of counting people in real time. In this proposal, the YOLO-PC obtained faster and more accurate results.

$YOLO_{V2}$ has been used in a real-time object detection system [45]. However, during the detection process, information about pedestrians was lost, which caused inaccurate pedestrian detection. Thus, in [50], the YOLO-R was proposed: an improvement on the $YOLO_{V2}$ structure. However, these studies have only focused on pedestrian detection.

Errors and omissions occur in the detection of pedestrians in one-stage algorithm models. However, in [51], an improved detection method based on $YOLO_{V3}$ was proposed. The study improved the human analysis and the overhang area in real time to detect pedestrians. In this proposal, the authors had two aims: extract more distinct attributes with human research and promote more real-time detection of regions through surveillance cameras.

Pedestrian detection is an important concern. However, due to the fact that the traffic of large urban centers is quite complex and changeable, many works focus on vehicle detection, and performing real-time detection is always a challenge. In [47], a method of real-time detection of vehicles and traffic lights was proposed. The method uses $YOLO_{V3}$, and a new dataset is created, called vehicle and traffic light (V-TLD), with the aim to improve the accuracy of vehicles. The one-stage DL algorithm model, such as YOLO, has been widely used in applications for the real environment, as it has lower requirements compared to other two-stage algorithms such as Faster R-CNN [11]. However, the accuracy and short running time is also a challenge in real-time traffic.

Detecting traffic inflation automatically is also a concern in intelligent traffic systems. Thus, some [52] have proposed an inflation detection method using two methods of object detection: $YOLO_{V3}$ and algorithms based on CNN. $YOLO_{V3}$ was used to detect motorcyclists, and the CNN was used to check whether or not the person was wearing a helmet. The model achieved a rate of 96.23% in accuracy for helmet detection. $YOLO_{V3}$ was also used in [53], reaching an 86% accuracy for vehicle detection. In [54], a system capable of detecting and tracking vehicles using $YOLO_{V3}$ was proposed. In the tests, it reached an mAP of 92.11% in vehicle counting on congested roads at a speed of 2.55 FPS. In [55], the authors proposed vehicle detection in real time using $YOLO_{V3}-live$, which is an enhanced version of $YOLO_{V3}-tiny$, reaching an mAP equal to 87.79%.

According to [56], CNN-based two-stage object detection algorithms such as R-CNN [10], Fast R-CNN [38], and Faster R-CNN [11] are very accurate models. However, acquiring the detection speed is quite time-consuming, and this is not very feasible for real-time applications. One-stage detection models such as SSD [13] and YOLO [12] are faster at detecting objects in images, but with a relatively low accuracy rate compared to two-stage models.

In the literature, there are several proposals for improving one-stage algorithms such as $YOLO_{V3}$ [46], always aimed at improving the accuracy rate. In some proposals, the one-stage algorithms can even overcome the precision rates of two-stage algorithms, such as $YOLO_{V3}-tiny$ [57].

It is very difficult to determine what is the best current object detection system [40], as each one has its application and particularities. Thus, among applications with a focus on precision and not on detection time, the CNN-based two-stage algorithms are more recommended [58]. However, in real-time applications, one-stage algorithms such as SSD and YOLO are recommended for use, due to their processing speed [59]. It is important to note that, in our work, precision and processing speed are both of concern. Thus, $YOLO_{V3}$ was the algorithm chosen to be improved upon in this work because it has presented superior detection performance and has been successfully applied in many fields [60,61,62].

### 2.3. Traffic Light Solutions Using Artificial Intelligence

AI algorithms have been used for applications on traffic lights. Some proposals for smart traffic lights [63] use fuzzy logic, a subset of AI, for recognizing vehicles. However, sensors were used in [63] to signalize traffic lights. In [18], an AI system combined to IoT was implemented; in this system, cameras controlled traffic lights in a city. However, the study focused on pedestrians and not priority vehicles.

Some studies with a focus only on recognizing vehicles using images, with no sensors [64], are concerned only with determining the waiting time of every vehicle. Another traffic light proposal used AI [65], and the study focused only on obtaining a shorter waiting time for queues at intersections, based on the entry of vehicles at these intersections. In [66], a similar study was conducted: real-time video images from the cameras at intersections were used, and, based on traffic density, the solution changed the traffic lights through an algorithm in order to reduce traffic congestion. Another study [67] proposed a system to control the density of traffic in real time using digital image processing, obtaining better efficiency in general than existing systems. It is important to note that some studies [64,65,66,67] did not include priority vehicles.

In [68], an intelligent traffic light was proposed to avoid vehicle stops at intersections under low traffic conditions. It detected the presence of vehicles on the road using various input devices, such as radars, cameras, and sensors. However, the system was dependent on many devices to perform the communication. Currently, there are many proposals for traffic lights that use an IoT system with presence sensors. In [69], the Raspberry Pi [69] was combined with different sensors performing a fully automated traffic light, which changes the waiting time at the intersection. Meanwhile, a traffic light was proposed in [17] with the aid of audible sensors and traffic images, to give priority to safety and public health vehicles. However, the captured images used in the system worked with low accuracy and an intelligent traffic light through sensors, which can generate a higher cost due to the cost of these sensors.

Currently there are traffic light proposals that give priority to rescue vehicles [29,70]. However, the majority of them uses sensors as well as image detection and classification algorithms with low accuracy [17,29,70].

Our proposed PVIDNet algorithm is used with an improved version of $YOLO_{V3}$. The algorithm $YOLO_{V3}$ has shown high power in the speed of object detection in the literature in relation to other algorithms. However, our proposal aims to decrease the processing time of detecting objects in the image.

### 2.4. Brazilian Traffic Code

The current Brazilian Traffic Code (BTC) was approved in 1998 [71] and had a major impact on society after its implementation [72]. It was found to cause an 18.5% reduction in the number of accidents. The current BTC stipulates that pedestrians always have priority in traffic, as long as they use the pedestrian crossings. Health and public safety vehicles also have priorities as long as they are properly identified, with audible and visual warnings, such as the activation of sirens. According to Art. 29 of the Brazilian Traffic Code, Law 9503/97, paragraph VII [71], vehicles such as ambulances, police cars, and fire trucks must have free movement in public roads when they are performing emergency services and properly identified by the recommended devices. However, there is no regulation about an automatic mechanism that gives priority to emergency vehicles at traffic lights.

In this work, an algorithm to control traffic lights is proposed, with the aim to give the right of way to emergency vehicles such as ambulances, fire trucks, and police cars in urban traffic. To this end, the outputs of the proposed PVIDNet are used as the inputs of an urban traffic simulation scenario.

## 3. Methodology

In this section, the main steps followed in building the proposed intelligent traffic light solution are described. Firstly, the database used in this work is presented. Later, the Deep Learning algorithm in which the Lightweight Priority Vehicle Image Detection Network (PVIDNet) is introduced, and the performance validation metrics used in the tests are also treated. Finally, the proposed control algorithm for an Intelligent Traffic Light is presented, and simulation scenario configurations using the SUMO tool are included.

**Figure 1:**
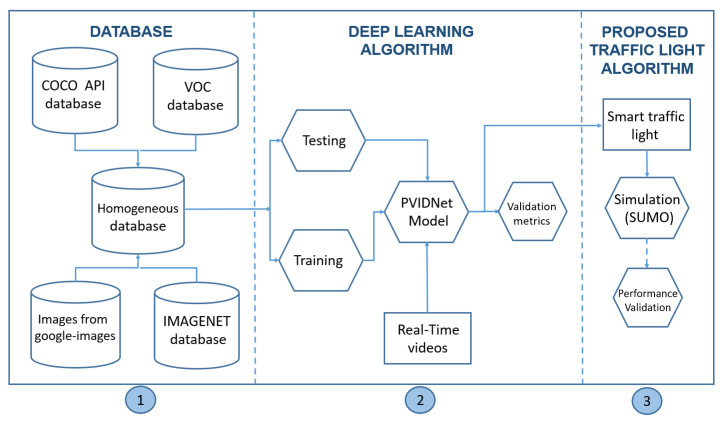
Flowchart of the proposed solution for the proposed intelligent traffic light.

Figure 1 illustrates how the proposed intelligent traffic light is developed, representing a general flowchart of the methodology, showing the three main steps involved in obtaining the proposed solution. In general, these three steps can be summarized as follows:

1. The Database. This is a set of homogeneous images used to train the DL model. The set of images is extracted from the following databases: COCO API, VOC, Google images, and IMAGINET.
2. Development of the Deep Learning Algorithm. This contains the Training and Testing phases to obtain the proposed model, PVIDNet. It is important to note that, in the validation step, a video about urban traffic is used; in this step, identification and classification of each vehicle is performed. For the performance validation assessment of the PVIDNet algorithm, the validation metrics, accuracy, sensitivity, and F-measures are used.
3. The Proposed Traffic Light Algorithm. After the classification of the vehicles by the DL algorithm, the traffic light controller works according to the vehicle’s priority proposed in this work. For evaluating the improvement of the traffic operation using the proposed traffic light algorithm, a simulation scenario is implemented with the SUMO tool.

The steps are explained in more detail in the following.

### 3.1. Database

Due to the difficulty of finding a DB with images of different vehicle types with similar image characteristics, a new DB was built to carry out training and tests for the proposed solution. To accomplish this aim, four distinct DBs were used: Image-net [73], PASCAL VOC [74], Coco-API [75], and Google Images.

The DB used in this work is composed of five image classes: ambulances, fire trucks, police cars, buses, and regular cars. Each image class is subdivided into three subcategories: right, left, and front, which represent the angles of the image. In total, this DB is composed of 5250 distinct color images that are subdivided into 350 images to the right, 350 to the left, and 350 images from the front, reaching a total of 1050 images for each class.

**Figure 2:**
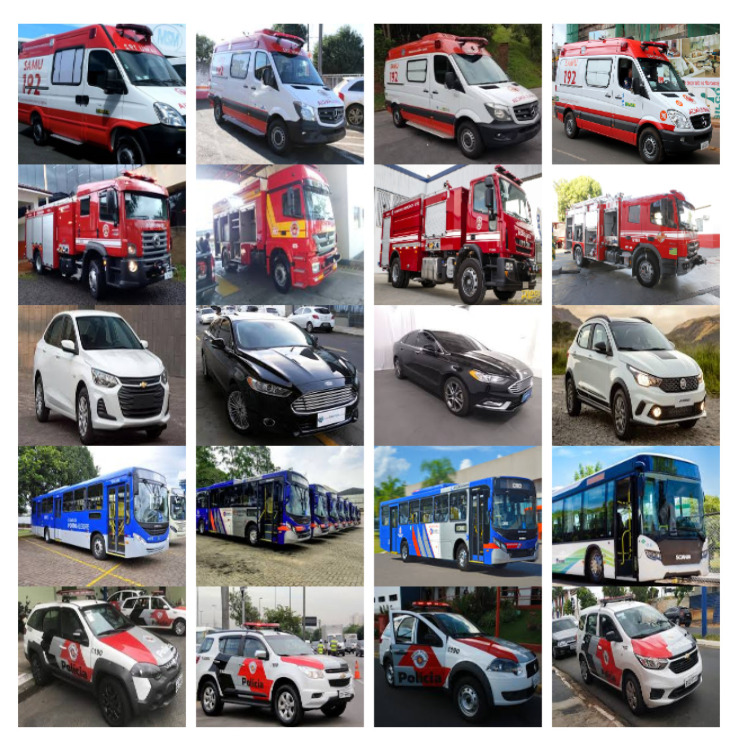
Database images created and their subcategories (right).

**Figure 3:**
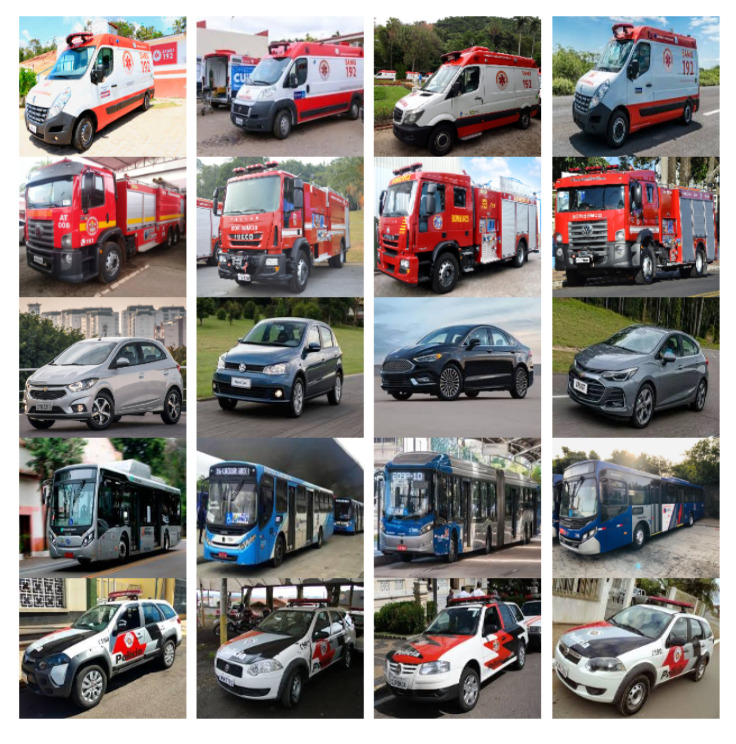
Images of the database created and its subcategories (left).

**Figure 4:**
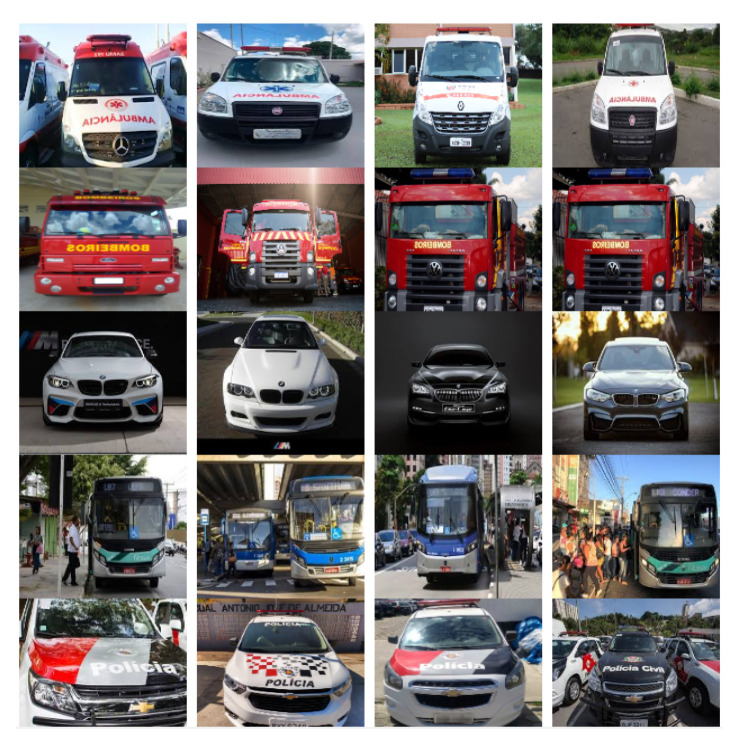
Database images created and their subcategories (front).

The resolution of each image was normalized to 1280 × 720. The following images from the DB in Figure 2 represent the right subcategory. Figure 3 represents the left subcategory, and Figure 4 represents the front subcategory.

**Figure 5:**
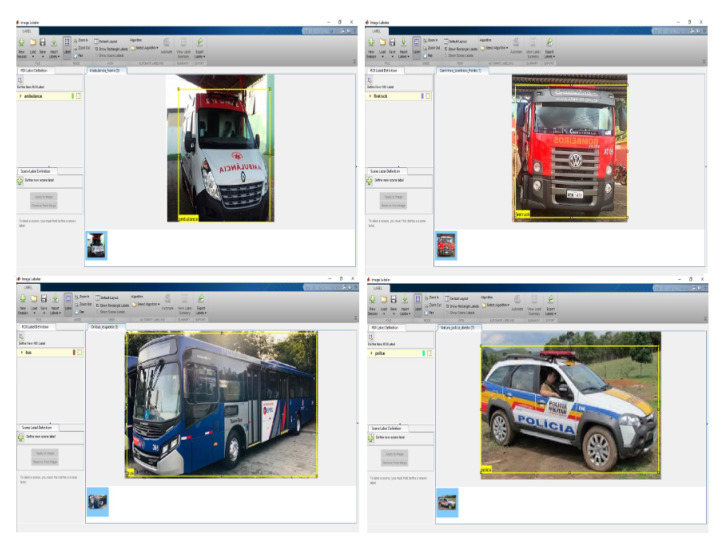
Images of the database created with standard Brazilian vehicles.

After creating the database, it is necessary to select the region of interest of each image that is being trained. The tool used was the Image Labeler from Matlab2019. Figure 5 illustrates images of the BD in which the Image Labeler was used.

The coordinates of the images used in this work are presented in Table 1.

### 3.2. Development of the Proposed Deep Learning Algorithm

As previously stated, the proposed PVIDNet solution used in this work is an improved version of $YOLO_{V3}$ [46].

Algorithm 1 represents the steps for performing object detection. Initially, the program is started together with the variables. After performing this process, the created database is loaded. The database is divided into 80% for training and 20% for testing. The training options, such as the number of interactions, times, learning rates, learning factor, speed, and penalty limit, are defined. After executing these processes, the command for training the network is executed. After training and testing of the database, the created network model is tested with urban traffic videos.
**Algorithm 1** Algorithm for Object Detection of the Proposed Solution.1:Load data from DB;2:Split the DB3:Determine the size of the incoming network4:Change the images to the size of the network entry5:Read the training data6:Select training options7:Train the network8:To evaluate the network with the test data9:To validate the network with urban traffic videos

PVIDNet was organized to detect objects at various scales, and it also needs resources of those various scales. Consequently, the last three residual blocks will all be used for further detection.

PVIDNet is implemented using the Tensor-flow framework with Keras, and the detection models are trained and tested using an NVIDIA Titan X server. Table 2 presents the network initialization parameters used in this work.

In order to adapt the input required for PVIDNet, the input images must be adjusted to 416 × 416 pixels. The batch size used in this work is equal to 8. The adaptive moment estimation is based on [76], and was used to update the weights of the networks. The parameters, such as the initial learning rate, weight decay regularization, and momentum are the original parameters used in the original YOLOv3. The transfer learning is based on [77].

#### 3.2.1. Lightweight Priority Vehicle Image Detection Network (PVIDNet)

In this work, a feature concatenation strategy is proposed. The feature maps learned from each image block are concatenated to all subsequent blocks. These blocks are used as inputs through pooling. Thus, the feature maps of all block outputs are concatenated together, in the backbone network, as inputs to the detection module. Through feature reuse and propagation, the input feature maps present an enriched representation power. The map provides additional information for characteristic learning, and this is defined in Equation (1).

(1) $$VehIBlock_{i}=Conc\left[MaxP\left(VehOBlock_{1},\;VehOBlock_{2},\;\ldots ,VehOBlock_{i-1}\right)\right]$$

where VehIBlocki and VehOBlocki represent the input and the output feature maps in the backbone network, respectively. The MaxP variable represents the max-pooling operation, and the Conc variable represents the concatenation operation.

A batch normalization layer and the SRS are used in the network. They are used for dimension reduction and accelerating convergence. The SRS can adjust the output through a pair of independent trainable parameters, presenting a better generalization performance and a faster learning speed.

The SRS activation function is defined in Equation (2).

(2) $$SRS\left(t\right)=\frac{t}{\frac{t}{\alpha }+e^{-\frac{t}{\beta }}}$$

where the α and β variables represent a pair of trainable positive parameters. The SRS represents a non-monotonic region in which t<0 provides the zero-mean property. When t>0, it avoids and rectifies the output distribution. The SRS derivative is defined in Equation (3).

(3) $$SRS^{\prime }\left(t\right)=\frac{\left(1+\frac{t}{\beta }\right)e^{-\frac{t}{\beta }}}{{\left(\frac{t}{\alpha }+e^{-\frac{t}{\beta }}\right)}^{2}}$$

The SRS is bounded output, presenting the range $\frac{\alpha \beta }{\beta -\alpha e},\alpha$.

In the experiments, Softmax and RELU were tested for comparison.

**Figure 6:**
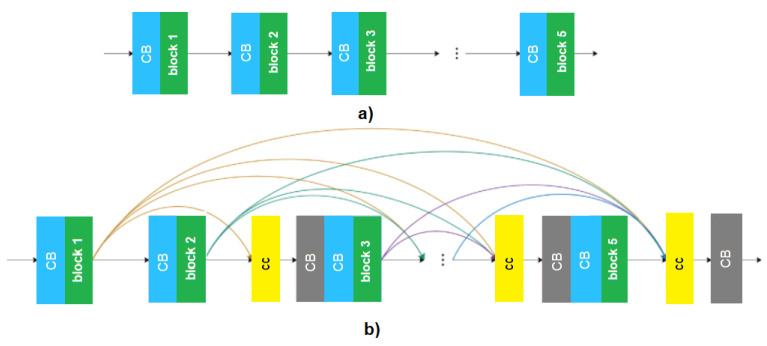
(**a**) representation of the backbone structure of the original YOLOv3; (**b**) representation of the backbone structure of PVIDNet.

The original YOLOv3 model presents several residual blocks, and this fact brings a large number of parameters to the network. Many parameters lead to an extended time training and slow down the detection speed of the model. Thus, the structure of PVIDNet needs to be optimized for real-time working. In PVIDNet, a modification in YOLOv3 was performed, as shown in Figure 6.

The Dense Block solution [22] presents some advantages, such as computational and storage efficiency. For this reason, it is used in PVIDNet. DenseNet needs only half of the parameters of the network for the same prediction accuracy, decreasing the complexity of the model and accelerating the detection of the vehicles. This approach yields a good image feature learning ability for PVIDNnet, and improves vehicle detection accuracy.

The dense connection structure of the convolutional layers, i.e., the Dense Connection blocks, are used for replacing the residual blocks located in the PVIDNet backbone network.

Each layer of the Dense Connection block outputs *m* feature maps, which represents the growth rate. The i-th layer of the block is represented by m0+m×(i−1). It is concatenated as an input. The number of the input feature maps of the first layer is represented by m0.

The Dense Connection block presents five densely connected units, as shown in Figure 6. Figure 6a shows the representation of the backbone structure of the original YOLOv3. In Figure 6b, each unit has a 1 × 1 convolutional layer represented by the grey color with label CB, which means Convolution-Batch Normalization with an activation function. Each unit also has a 3 × 3 convolutional layer represented by the blue color in Figure 6b, in which each convolutional layer is followed by a batch normalization layer and the SRS activation function. The yellow block named cc in Figure 6b represents the feature concatenation.

In this network, the *m* growth rate is set to 32. Improved transition blocks are used before each Dense Connection block, for performing the maximum pooling and convolution step. In the end, it concatenates both outputs as being the input of the next block. Thus, the overall parameters of the new network are reduced; therefore, the processing time is also decreased.

It is important to note that the Dense Connection block effectively smooths the strengthen feature propagation, the gradient vanishes, and feature reuses are facilitated. This block differentiates the received data that is added to the network and preserves it. Thus, the network knowledge is held, helping to base decisions on all feature maps of the network. This process makes the proposed system applicable in real-time scenarios.

In the experiments, the proposed PVIDNet model using the SRS activation function is compared with YOLOv3, PVIDNet using Softmax, and PVIDNet with Relu.

#### 3.2.2. Validation with Real-Time Videos

For validation, video vehicles in real-time and in real scenes were captured. Fifty videos were recorded. The length of each collected video was 40 s, a value chosen according to related studies [78]. The videos are 34.25 FPS and were captured with an EOS 550D camera at four different locations, under three occlusion statuses. It is important to note that the videos had all types of vehicles considered in this study: ambulances, police cars, fire trucks, buses, and regular cars.

#### 3.2.3. Model Validation Metrics

In this work, the following validation metrics are used: accuracy, sensitivity or recall, and F-Measure. These metrics are composed of true positive (TP), false positive (FP), false negative (FN), and true negative (TN).

These metrics are defined as follows:

- (4) $$Accuracy=\frac{TP+TN}{TP+TN+FN+TN}$$
- (5) $$Sensitivity\;or\;Recall=\frac{TP}{TP+FN}$$
- (6) $$F-Measure=2*\frac{Precision*Recall}{Precision+Recall}$$
- Precision is defined as follows:
  (7) $$Precision=\frac{TP}{TP+FN}$$

In this work, 10-fold cross-validation is performed to obtain the metrics for the validation of the vehicle classification.

### 3.3. The Proposed Traffic Light Control Algorithm

After the vehicles are classified by PVIDNet, which performs faster detection based on its activation function and feature concatenation, the proposed traffic light control algorithm is applied. Thus, the outputs of the proposed deep learning algorithm are used as inputs for the traffic light control algorithm based on vehicle priorities, in order to make a decision about the traffic flow.

**Figure 7:**
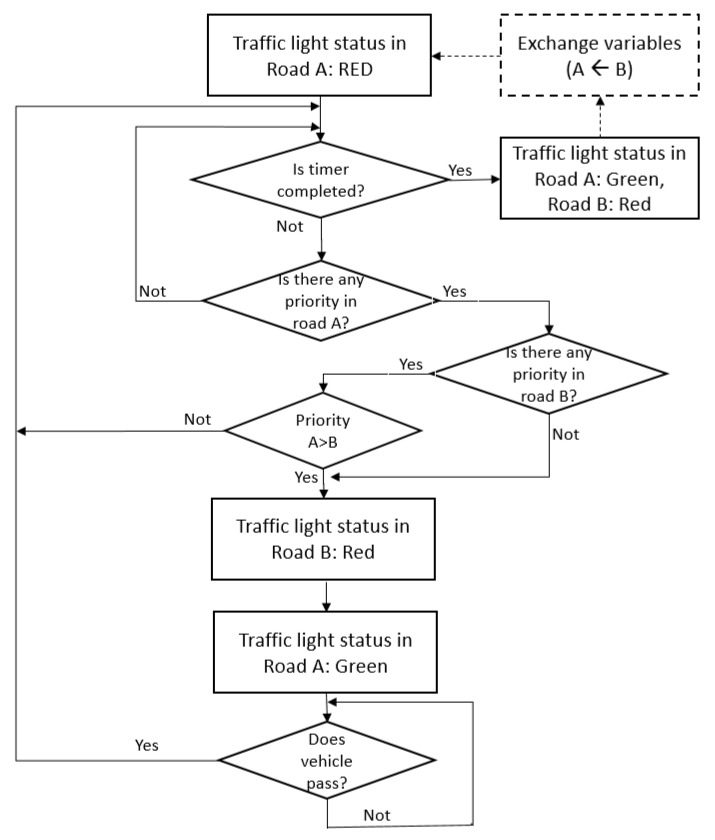
The proposed control algorithm for an intelligent traffic light.

The proposed traffic light control algorithm works according to Figure 7. It is formulated based on BTC [71] but considering some adaptations regarding the priority of emergency vehicles.

Initially, in the proposed algorithm for an intelligent traffic light shown in Figure 7, the traffic light on Road A is considered as being red. Note that the analysis is only performed on a traffic light status that is red or green, and a traffic light status of yellow is not considered in the diagram for simplicity. When the traffic light timer is completed, then the traffic light status on Road A is set to green, and the traffic light status on Road B is set to red as a regular traffic light based on the timer. When the timer is not completed, the preference or right of way of priority vehicles is verified; when a priority vehicle appears on Road A, then the traffic light on Road B is set to red, and the traffic light on Road A is set to green. It is important to note that a priority index of vehicles is followed in this work; for instance, ambulances have a higher priority than regular cars. When the priority vehicle passes the traffic light on Road A, then the flow restarts with the verification if the timer is completed. The same logic occurs with the traffic light on Road B; for this purpose, the block “A←B” shows a change of variables.

The traffic simulation scenarios show that different vehicles can be present together at the same time in a road intersection controlled by a traffic light. These vehicles are identified, and the priority order of each one is obtained considering the traffic light priority presented in Table 3. When vehicles of the same class are detected on both roads, and this class is the highest priority at that moment in that road intersection, the vehicle in the road with the traffic light status of green has the right of way in terms of crossing the road.

In the proposed solution, the control traffic light gives the right of way to priority vehicles traveling on the road. Thus, the traffic light manages this automatically, and there needs to be a communication between both traffic lights. These vehicles are emergency vehicles, such as ambulances and fire trucks. Among these vehicles, the order of priority chosen in this work is as follows: ambulance, fire truck, police car, bus, and regular car, respectively.

The priority index chosen in this work is shown in Table 3, in which 0 represents the highest priority, and 4 represents the lowest priority. The index is used to detect emergency vehicles approaching the intersection. Thus, emergency vehicles have the highest priority, while a non-emergency vehicle such as a car has the lowest priority.

When a priority vehicle is detected by the traffic light controller, its status is changed to the green phase, and the controller of the traffic light extends the duration of this phase until the priority vehicle passes through the intersection. It is worth noting that, in our experimental studies, ambulances, fire trucks, and police cars are considered as emergency vehicles, and they have the highest priorities.

The scenario chosen to be simulated is implemented in the Simulation of Urban Mobility (SUMO) [70], which is an Open Source road traffic simulator with a realistic road topology.

#### Simulation Configuration with a SUMO Tool

To evaluate the improvement of traffic operation, the SUMO is used in this work. The proposed algorithm for an intelligent traffic light was simulated and compared with the FIFO strategy.

The identification of priority vehicles is performed by the traffic light. Thus, when a vehicle of high priority appears in the scenario, the traffic light produces a prioritized green light on demand to allow the vehicle to immediately pass intersections. The SUMO traffic light turns green in the scenario through a variable.

The simulations were carried out using the same environment, and the parameters are presented in Table 4. The behavior of the traffic arrival rates follows a Poisson distribution.

The average total waiting time and total travel time (TTT) were used to evaluate the performance of the methods. The expected average speed was generated based on the Greenshields model [79]. The FIFO strategy was selected for comparison because the related studies do not present a similar system that can be used for comparison.

**Figure 8:**
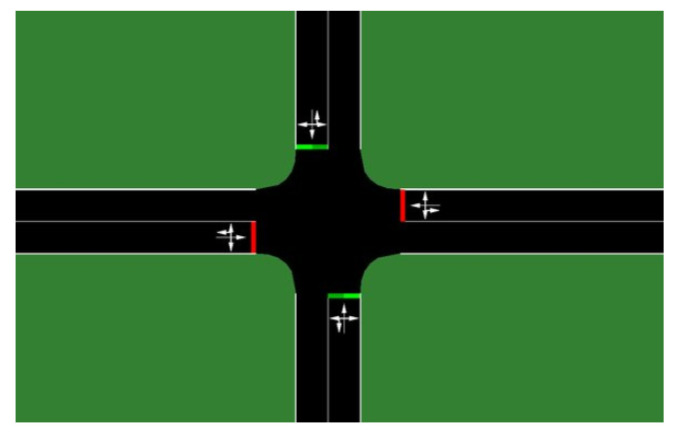
A simple intersection scenario in the SUMO, used in the experiments.

The simulation map used in this work is based on a four-leg road intersection, as shown in Figure 8. A roadside unit is positioned at the intersection. In the map used, all the roads have two lanes in the same traffic direction with a total length of 1 km upstream and downstream.

The emergency vehicles, including ambulances, fire trucks, and police cars, make up 5% of all vehicles and are entered into the simulation with a Poisson arrival rate.

## 4. Results and Discussion

In the experiments, the proposed model, PVIDNet, is compared to YOLOv3, and the proposed solution has different activation functions. The performance performance of the proposed intelligent traffic light solution composed of both PVIDNet and the traffic control algorithm is compared with the FIFO strategy in the SUMO tool, using video sequences.

### 4.1. Evaluation of the Proposed System, PVIDNet, with the Image Database

Table 5, Table 6, Table 7, Table 8 and Table 9 present the performance assessment results of the proposed PVIDNet with different activation functions in relation to YOLOv3 to classify the images in the training phase for each class of vehicle. These results correspond to each of the angles of the image.

Table 10 presents the performance assessment results of our proposal considering a dataset composed by the right, left, and front images.

Table 11, Table 12, Table 13, Table 14 and Table 15 present the performance assessment results of the proposed PVIDNet with different activation functions in relation to YOLOv3 to classify the images in the testing phase for each class of vehicle. These results correspond to each of the angles of the image.

Table 16 presents the performance assessment results of our proposal considering a dataset composed by the right, left, and front images.

In relation to the training and testing times, PVIDNet using the SRS activation function presents a time processing reduction of about 30% in relation to the time spent by the YOLOv3 algorithm.

### 4.2. Validation of the Proposed PVIDNet Using Video Data

In order to perform a validation assessment of PVIDNet using the SRS algorithm, simulation tests using video streams were performed. A total of 50 urban traffic videos were captured for experiments. The test results are shown in Table 17. It is noted that the accuracy rates of PVIDNet with the SRS algorithm are higher than those of the YOLOv3 algorithm.

As can be observed from Table 17, the results obtained are similar to the high accuracy values obtained using the DB of the images.

### 4.3. Validation of the Proposed Traffic Control Algorithm for Intelligent Traffic Lights

**Figure 9:**
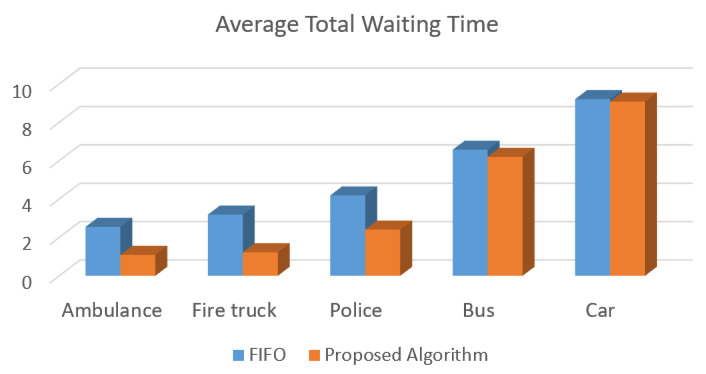
Average total waiting time reached by the proposed traffic control algorithm and FIFO for each type of vehicle.

Figure 9 presents the simulation results performed in the SUMO regarding the average of the total waiting time reached by the proposed solution and the FIFO algorithm. The proposed traffic control algorithm for intelligent traffic lights presented in Figure 7 reduces a great amount of the delay, reducing on average 50% of the waiting time for emergency vehicles. In the case of public transportation vehicles, the waiting time reduction was close to 5%, and, for regular cars, the waiting time was almost the same.

**Figure 10:**
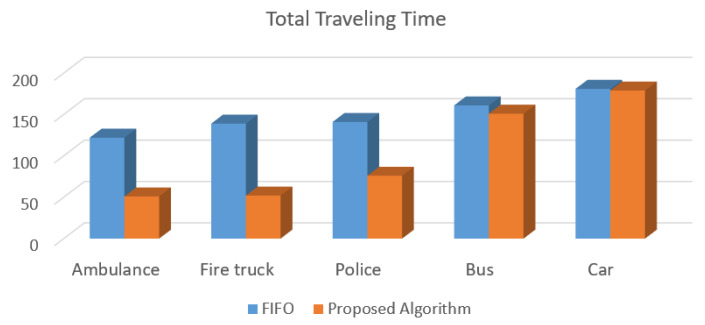
Total traveling time reached by the proposed traffic control algorithm and FIFO for each type of vehicle.

Figure 10 presents the simulation results performed in the SUMO of the total traveling time reached by the proposed solution and the FIFO algorithm. The proposed traffic control algorithm for intelligent traffic reduces on average 45% of the delay for emergency vehicles, and for the other vehicles the delay is almost the same when compared with the FIFO-based method.

As depicted in Figure 9 and Figure 10, the proposed control algorithm for intelligent traffic lights improves the time response of emergency vehicles, and regular vehicles are not negatively impacted in terms of travel time.

## 5. Conclusions

In this research, a priority vehicle image detection model was studied and implemented. Methods regarding image feature extraction and function activation in DL were investigated and evaluated. In addition, a DB was created, and it is composed of five types of vehicles, considering the left, frontal, and right angles of the image capture.

For improving the detection procedure and time execution, an improved version of YOLOv3 is proposed. Additionally, the incorporation of an improved version of DenseNet reduced the parameter numbers used by PVIDNet, enhancing feature propagation and network performance. The SRS activation function presented a low processing time compared to other functions because the SRS presents a better generalization performance and a faster learning speed for the model generation; thus, the deep network training process is accelerated. Performance assessment results demonstrated that the PVIDNet model reached an accuracy higher than 0.95 in vehicle image classification as presented in Table 16, and results are better for emergency vehicles. Furthermore, when the proposed model is validated using video sequences, the same high accuracy is reached, as presented in Table 17. Based on the BTC, a control traffic algorithm that gives priority to emergency vehicles, such as ambulances, fire trucks, and police cars, is proposed. For the performance assessment of the control algorithm, simulation tests were performed. To this end, the SUMO tool for simulating the traffic of vehicles was used. The simulation test results showed a decrease of 50% in the average total waiting time for emergency vehicles when compared to the FIFO strategy. Moreover, a decrease of 45% in total traveling time for emergency vehicles was achieved. It is important to note that regular private cars are not negatively affected regarding the waiting time and total travel time. In the case of public transportation, represented by buses, a slight improvement in those same parameters was obtained.

The experimental results demonstrated that the proposed solution composed of the Lightweight PVIDNet and a control algorithm for intelligent traffic presented a high accuracy with a low complexity, as well as a fast image detection process, which are important features of intelligent traffic lights. Furthermore, the reduction of waiting time at traffic lights for emergency vehicles is obviously important in an emergency situation.

In future work, we intend to explore other simulation scenarios, with other road intersections and vehicular traffic models. Additionally, we intend to develop a prototype of both the proposed PVIDNet model and the traffic light controller using embedded systems. 

## Figures and Tables

**Table 1:** Coordinates of the images.

Figure	Coordinates
a	47,41,331,361
b	47,41,331,361
b	47,41,331,361
d	1,18,282,151

**Table 2:** Initialization parameters used in PVIDNet.

Batch	Momentum	Learning Rate	Decay
8	0.9	0.01	0.005

**Table 3:** Traffic light priority.

Priorities	Vehicles
0	Ambulances
1	Fire trucks
2	Polices
3	Buses
4	Cars

**Table 4:** Simulation parameters used in the SUMO.

Variable Name	Value	Definition
Vehicles/two-lane road	500/h	number of vehicles
$x_{bn}$	700(m)	Position of the rear of bottleneck in the lane
$v_{max}$	120 km/h	Maximum speed of the vehicle
αmax	5 m/s${}^{2}$	Maximum acceleration
minGap	5(m)	Minimum space between two vehicles
Δt	0.1(s)	Time step length
Duration	70 min	Simulation duration

**Table 5:** Results of the DL algorithms to classify the images of fire trucks (right/left/front) in the training phase.

Model	Accuracy	Sensitivity	F-Measure
YOLOv3	0.88/0.87/0.88	0.87/0.88/0.87	0.87/0.87/0.87
PVIDNet (Softmax)	0.91/0.90/0.90	0.89/0.90/0.89	0.89/0.89/0.89
PVIDNet (Relu)	0.93/0.91/0.92	0.92/0.91/0.92	0.92/0.91/0.92
PVIDNet (SRS)	0.95/0.95/0.94	0.94/0.94/0.95	0.94/0.94/0.94

**Table 6:** Results of the DL algorithms to classify the images of buses (right/left/front) in the training phase.

Model	Accuracy	Sensitivity	F-Measure
YOLOv3	0.86/0.87/0.87	0.85/0.86/0.86	0.85/0.86/0.86
PVIDNet (Softmax)	0.89/0.89/0.90	0.88/0.90/0.88	0.88/0.89/0.88
PVIDNet (Relu)	0.91/0.91/0.92	0.91/0.91/0.92	0.91/0.91/0.92
PVIDNet (SRS)	0.94/0.93/0.94	0.94/0.93/0.95	0.94/0.93/0.94

**Table 7:** Results of the DL algorithms to classify the images of the ambulances (right/left/front) in the training phase.

Model	Accuracy	Sensitivity	F-Measure
YOLOv3	0.85/0.84/0.85	0.85/0.85/0.84	0.85/0.84/0.84
PVIDNet (Softmax)	0.87/0.87/0.88	0.88/0.88/0.87	0.87/0.87/0.87
PVIDNet (Relu)	0.91/0.90/0.92	0.89/0.90/0.90	0.89/0.90/0.90
PVIDNet (SRS)	0.93/0.94/0.94	0.93/0.93/0.93	0.93/0.93/0.93

**Table 8:** Results of the DL algorithms to classify the images of police cars (right/left/front) in the training phase.

Model	Accuracy	Sensitivity	F-Measure
YOLOv3	0.83/0.84/0.83	0.82/0.83/0.82	0.83/0.84/0.83
PVIDNet (Softmax)	0.86/0.86/0.87	0.86/0.87/0.86	0.86/0.87/0.87
PVIDNet (Relu)	0.90/0.89/0.91	0.89/0.90/0.90	0.90/0.90/0.90
PVIDNet (SRS)	0.92/0.93/0.93	0.92/0.93/0.93	0.92/0.93/0.93

**Table 9:** Results of the DL algorithms to classify the images of regular cars (right/left/front) in the training phase.

Model	Accuracy	Sensitivity	F-Measure
YOLOv3	0.82/0.81/0.81	0.81/0.81/0.82	0.81/0.81/0.81
PVIDNet (Softmax)	0.85/0.84/0.85	0.85/0.85/0.86	0.85/0.84/0.85
PVIDNet (Relu)	0.89/0.88/0.89	0.89/0.88/0.89	0.89/0.88/0.89
PVIDNet (SRS)	0.92/0.92/0.92	0.92/0.92/0.93	0.92/0.92/0.92

**Table 10:** Accuracy results of the machine learning algorithms classifying the images of Fire Trucks, Buses, Ambulances, Police Cars, and Regular Cars in the training phase considering together the right, left, and front images.

Model	Fire Truck	Bus	Ambulance	Police Car	Regular Car
YOLOv3	0.90	0.88	0.87	0.85	0.84
PVIDNet (Softmax)	0.92	0.91	0.89	0.87	0.86
PVIDNet (Relu)	0.93	0.92	0.91	0.89	0.88
PVIDNet (SRS)	0.98	0.98	0.96	0.95	0.95

**Table 11:** Results of the DL algorithms to classify the images of fire trucks (right/left/front) in the testing phase.

Model	Accuracy	Sensitivity	F-Measure
YOLOv3	0.87/0.87/0.88	0.87/0.86/0.87	0.87/0.87/0.87
PVIDNet (Softmax)	0.90/0.90/0.90	0.89/0.90/0.89	0.90/0.89/0.89
PVIDNet (Relu)	0.93/0.92/0.92	0.92/0.91/0.92	0.92/0.92/0.92
PVIDNet (SRS)	0.95/0.94/0.94	0.94/0.94/0.95	0.94/0.94/0.94

**Table 12:** Results of the DL algorithms to classify the images of buses (right/left/front) in the testing phase.

Model	Accuracy	Sensitivity	F-Measure
YOLOv3	0.86/0.86/0.87	0.85/0.86/0.86	0.85/0.86/0.86
PVIDNet (Softmax)	0.90/0.89/0.90	0.88/0.90/0.88	0.89/0.89/0.88
PVIDNet (Relu)	0.90/0.91/0.92	0.91/0.91/0.92	0.90/0.91/0.92
PVIDNet (SRS)	0.93/0.93/0.95	0.94/0.93/0.95	0.93/0.93/0.95

**Table 13:** Results of the DL algorithms to classify the images of ambulances (right/left/front) in the testing phase.

Model	Accuracy	Sensitivity	F-Measure
YOLOv3	0.84/0.84/0.85	0.84/0.85/0.84	0.84/0.84/0.84
PVIDNet (Softmax)	0.87/0.87/0.88	0.87/0.88/0.87	0.87/0.87/0.87
PVIDNet (Relu)	0.91/0.90/0.92	0.89/0.90/0.90	0.89/0.90/0.90
PVIDNet (SRS)	0.93/0.94/0.93	0.93/0.93/0.93	0.93/0.93/0.93

**Table 14:** Results of the DL algorithms to classify the images of police cars (right/left/front) in the testing phase.

Model	Accuracy	Sensitivity	F-Measure
YOLOv3	0.82/0.84/0.83	0.82/0.83/0.82	0.82/0.84/0.83
PVIDNet (Softmax)	0.86/0.86/0.87	0.86/0.87/0.86	0.86/0.87/0.87
PVIDNet (Relu)	0.90/0.89/0.91	0.90/0.90/0.90	0.90/0.90/0.90
PVIDNet (SRS)	0.92/0.93/0.93	0.92/0.92/0.93	0.92/0.92/0.93

**Table 15:** Results of the DL algorithms to classify the images of regular cars (right/left/front) in the testing phase.

Model	Accuracy	Sensitivity	F-Measure
YOLOv3	0.79/0.81/0.81	0.80/0.81/0.82	0.79/0.81/0.81
PVIDNet (Softmax)	0.84/0.84/0.85	0.85/0.85/0.86	0.84/0.84/0.85
PVIDNet (Relu)	0.88/0.88/0.89	0.88/0.88/0.89	0.88/0.88/0.89
PVIDNet (SRS)	0.92/0.91/0.92	0.92/0.92/0.93	0.92/0.91/0.92

**Table 16:** Accuracy results of the DL algorithms classifying the images of Fire Trucks, Buses, Ambulances, Police Cars, and Regular Cars in the testing phase considering together the right, left, and front images.

Model	Fire Truck	Bus	Ambulance	Police Car	Regular Car
YOLOv3	0.89	0.87	0.87	0.84	0.84
PVIDNet (Softmax)	0.92	0.92	0.89	0.86	0.86
PVIDNet (Relu)	0.94	0.92	0.91	0.88	0.87
PVIDNet (SRS)	0.97	0.97	0.96	0.95	0.95

**Table 17:** Accuracy results classifying Fire Trucks, Buses, Ambulances, Police Cars, and Regular Cars using video stream data considering together the right, left, and front images.

Model	Fire Truck	Bus	Ambulance	Police Car	Regular Car
YOLOv3	0.86	0.86	0.85	0.83	0.81
PVIDNet (SRS)	0.98	0.97	0.96	0.96	0.95
